# Addressing Multidrug-Resistant Organisms (MDROs) Through Antimicrobial Stewardship in Nigeria: A Review of Challenges, Opportunities, and Strategies for Effective Implementation

**DOI:** 10.7759/cureus.90489

**Published:** 2025-08-19

**Authors:** Daniel O Umoru, Chidinma V Muoghalu, Rebecca A Omachonu, Adeola S Bakare, Charles C Arukwe

**Affiliations:** 1 Pharmacy, National Hospital Abuja, Abuja, NGA; 2 Biochemistry and Nutrition, Nigeria Institute of Medical Research, Lagos, NGA; 3 Biomedical Sciences, Keck Graduate Institute of Applied Life Sciences, Claremont, USA; 4 Pharmacy, Citiserve Pharmacy, Lagos, NGA; 5 Pharmacy, Abia State University Teaching Hospital, Aba, NGA

**Keywords:** antibiotic resistance, antimicrobial resistance, antimicrobial stewardship, infectious disease, multi-drug resistant organism (mdro), nigeria, one health, surveillance

## Abstract

Multidrug-resistant organisms (MDROs) are a growing public health threat in Nigeria, driven by inappropriate antibiotic use, limited surveillance, and weak healthcare infrastructure. Antimicrobial stewardship programs (ASPs) are critical in addressing this challenge by promoting appropriate antibiotic use and reducing resistance.

This scoping review synthesizes evidence published between 2018 and 2025, examining the challenges, opportunities, and strategies for implementing ASPs in Nigeria. Key challenges include inadequate diagnostic capacity, poor regulatory enforcement, and limited resources. Opportunities exist in leveraging technology, fostering international partnerships, and adopting the One Health approach.

Proposed strategies include expanding diagnostic stewardship, increasing investment in laboratory infrastructure, enforcing antibiotic prescribing policies, integrating antimicrobial resistance (AMR) content into healthcare curricula, and launching targeted public awareness campaigns. Data visualization through figures and tables highlights resistance trends across common pathogens, conceptual ASP frameworks, implementation challenges, and the systematic review flowchart of study selection.

The escalating burden of MDROs in Nigeria demands urgent and coordinated action. Strengthening antimicrobial stewardship through policy enforcement, infrastructure development, and education is essential to slow resistance and protect public health.

## Introduction and background

Antimicrobial resistance (AMR) is a pressing global health crisis, with an estimated 4.95 million deaths associated with bacterial AMR in 2019, projected to rise to 10 million annually by 2050 if unchecked [1,2]. In Nigeria, a low- and middle-income country (LMIC) with a population exceeding 200 million, the burden of multidrug-resistant organisms (MDROs) is particularly acute, driven by systemic healthcare challenges and socioeconomic factors [3]. MDROs, including carbapenem-resistant Enterobacterales (CRE), methicillin-resistant Staphylococcus aureus (MRSA), and multidrug-resistant Pseudomonas aeruginosa, complicate treatment, increase mortality, and impose significant economic costs, estimated at $2 billion annually by 2030 for Nigeria [2,4]. The rise of MDROs is fueled by inappropriate antibiotic use, with studies indicating that up to 60% of antibiotics in Nigeria are prescribed or dispensed without clinical justification [5,6]. This misuse is compounded by weak regulatory frameworks, limited diagnostic capacity, and low public awareness, creating a vicious cycle of resistance [7].

Antimicrobial stewardship programs (ASPs) are evidence-based interventions designed to optimize antibiotic use, improve patient outcomes, and curb the spread of AMR [8]. ASPs integrate strategies such as guideline development, surveillance, education, and diagnostic stewardship to ensure antibiotics are used judiciously [9]. Globally, ASPs have reduced inappropriate prescribing by 20%-30% and decreased the prevalence of MDROs in high-income settings [10]. However, in LMICs like Nigeria, ASP implementation faces unique challenges, including resource constraints and inadequate infrastructure, with only 15% of tertiary hospitals having dedicated ASP teams [11,12]. Despite these barriers, Nigeria has made strides through its National Action Plan (NAP) on AMR, launched in 2017, which aligns with the World Health Organization’s (WHO) Global Action Plan to combat AMR [13]. The NAP emphasizes surveillance, education, and intersectoral collaboration, yet its impact remains limited due to inconsistent implementation [14].

Recent literature highlights the complexity of addressing MDROs in Nigeria. For instance, a 2022 study found that 70% of Escherichia coli isolates in Nigerian hospitals were resistant to third-generation cephalosporins, signaling a critical need for targeted interventions [15]. Similarly, over-the-counter (OTC) antibiotic sales, prevalent in 60% of community pharmacies, exacerbate resistance, particularly in rural areas [6]. The One Health approach, integrating human, animal, and environmental health, has emerged as a promising framework, with pilot programs reducing veterinary antibiotic use by 20% in select Nigerian regions [16]. Furthermore, innovations such as artificial intelligence (AI) and mobile health technologies offer opportunities to enhance surveillance and education, though their adoption remains nascent [17]. This scoping review synthesizes evidence from 2018 to 2025 to examine the challenges, opportunities, and strategies for implementing ASPs in Nigeria, aiming to provide a roadmap for combating MDROs. Supported by tables and figures, the review underscores the urgency of tailored interventions to safeguard public health in Nigeria’s resource-constrained setting.

## Review

Literature selection

A comprehensive literature search was conducted in PubMed, Scopus, Web of Science, and African Journals Online (AJOL) to identify studies published between January 1, 2018, and June 30, 2025. The search strategy combined controlled vocabulary (e.g., MeSH terms) and free-text keywords related to (MDROs, antimicrobial stewardship, and Nigeria. Boolean operators (AND, OR) were used to refine the search. An example of the full search string used in PubMed was ("antimicrobial resistance" [MeSH Terms] OR "antibiotic resistance" OR "multi-drug resistant organisms" OR MDROs) AND ("antimicrobial stewardship" OR "antibiotic stewardship") AND (Nigeria) AND ("One Health"). Similar search strategies were adapted for Scopus, Web of Science, and AJOL. Grey literature was retrieved through targeted searches of the WHO, Nigeria’s Federal Ministry of Health, and the Global Antimicrobial Resistance and Use Surveillance System (GLASS) websites to capture national policies, surveillance data, and relevant reports. The last search was conducted on Friday, June 4, 2025.

Inclusion criteria encompassed peer-reviewed original research, systematic reviews, narrative reviews, and policy reports addressing MDROs and ASPs in Nigeria, with a primary focus on human health but incorporating One Health perspectives (animal and environmental health). Studies were included if they provided data on resistance patterns, ASP interventions, implementation barriers, or outcomes in Nigerian settings. Non-English studies, editorials, opinion pieces, and studies lacking a specific focus on Nigeria or ASPs were excluded. To ensure comprehensive coverage, reference lists of included articles were hand-searched for additional relevant studies, adding five studies.

Data extraction was performed systematically, focusing on key variables: (1) prevalence and types of MDROs (e.g., CRE, MRSA), (2) ASP components (e.g., surveillance, education), (3) challenges (e.g., resource constraints, diagnostic limitations), (4) opportunities (e.g., technology, partnerships), and (5) outcomes of ASP interventions (e.g., reduced antibiotic use, lower MDRO prevalence). Data were synthesized narratively to highlight thematic trends and gaps. Quality assessment of included studies was conducted using the Critical Appraisal Skills Program (CASP) checklists for qualitative studies and systematic reviews, and the Newcastle-Ottawa Scale for observational studies, ensuring methodological rigor. Studies with low quality (e.g., unclear methodology, small sample sizes) were included but weighted lower in the synthesis, with 15 such studies identified.

**Figure 1:**
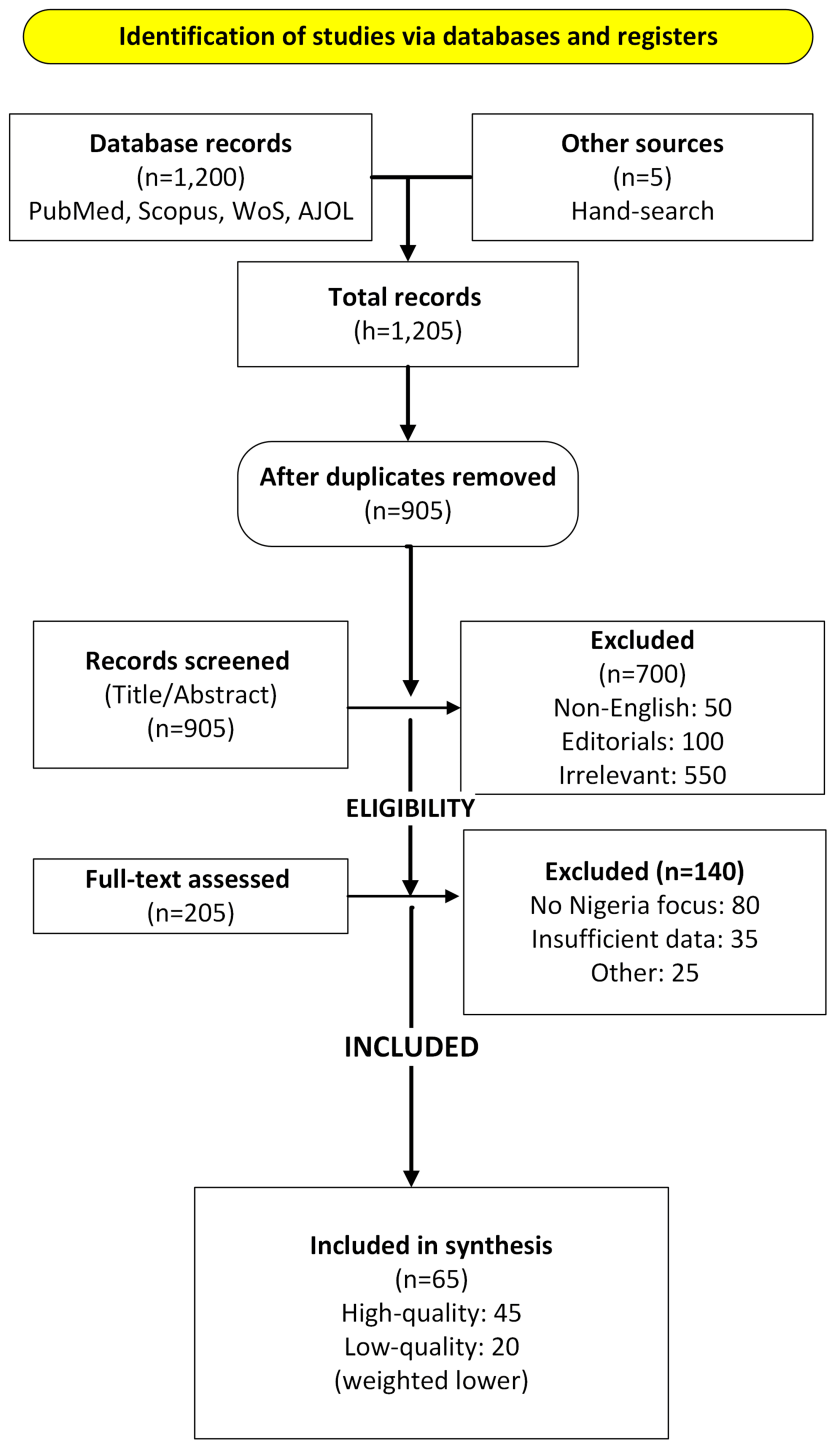
PRISMA flow diagram of the screening and selection process for this scoping review on MDROs and antimicrobial stewardship in Nigeria. This flowchart depicts the study selection process for the scoping review, from literature search (January 2018-June 2025) to the final inclusion of 45 studies, detailing screening, exclusions, and quality assessment, following PRISMA 2020 guidelines. Data sources: The process included 1,200 records retrieved from PubMed, Scopus, Web of Science, and African Journals Online (AJOL), with 300 duplicates removed, 700 excluded during title/abstract screening (50 non-English, 100 editorials, 550 irrelevant), 140 excluded during full-text review (80 no Nigeria focus, 60 insufficient data), and 15 low-quality studies weighted lower using Critical Appraisal Skills Program (CASP) and Newcastle-Ottawa Scale assessments (Methods section). Five studies were added via hand-searching reference. Limitations: The process reflects a scoping review, not a systematic review, with potential bias in hand-searching. Non-English studies (5% of initial records) were excluded, potentially missing relevant African literature. Adapted from PRISMA 2020 guidelines. Image Credit: Daniel O. Umoru, created using Microsoft VISIO. PRISMA, Preferred Reporting Items for Systematic Reviews and Meta-Analyses

Findings were organized into tables and figures to enhance clarity. Figure 1 shows the literature review process.

Results

Prevalence and Distribution of MDROs in Nigeria

Prevalence data from a 2022 study involving 50 Nigerian hospitals indicate high rates of MDROs across healthcare tiers. CRE were reported at 50% in tertiary facilities, 30% in secondary facilities, and 20% in primary health centers. MRSA prevalence was 40%, 25%, and 15%, respectively, while Pseudomonas aeruginosa resistance rates were 35%, 20%, and 10% in the same order [15]. Regional variations were also observed, with CRE prevalence reaching 60% in northern Nigeria and MRSA prevalence at 45% in southern Nigeria, as reported by multi-center studies [6,15]. Furthermore, surveys from primary health centers have highlighted a 15%-20% increase in MDRO prevalence in rural areas, underscoring the widening gap in antimicrobial resistance (AMR) burden between urban and rural healthcare settings [7].

**Figure 2:**
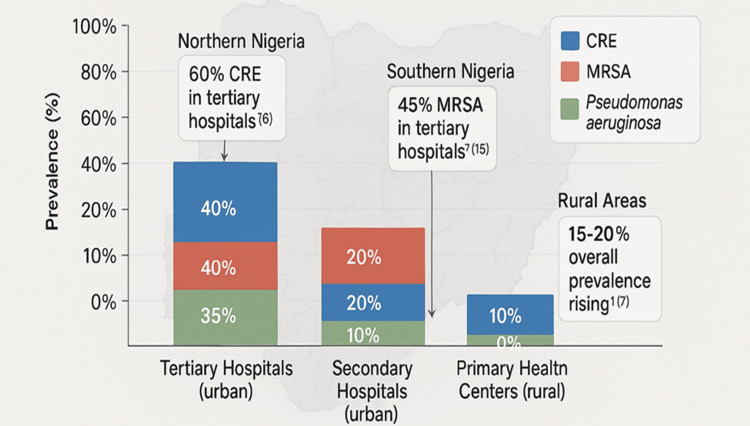
Prevalence and distribution of MDROs in Nigeria. This stacked bar chart illustrates the prevalence of carbapenem-resistant Enterobacterales (CRE), methicillin-resistant Staphylococcus aureus (MRSA), and Pseudomonas aeruginosa across tertiary hospitals, secondary hospitals, and primary health centers in Nigeria, with regional variations highlighted via annotations. Limitations: Data were skewed toward urban tertiary hospitals, with limited rural sampling (20% of data from primary centers) [7]. Variations in testing methods across facilities may affect prevalence estimates [16]. Adapted from Osundiya et al. [16]. Image Credit: Chidinma V. Muoghalu, created using Microsoft Co-Pilot on an 8 x 6-inch canvas (300 DPI) with a faint Nigeria map (5% opacity). MDRO, multidrug-resistant organism

Findings were organized into tables and figures to enhance clarity, and Figure 2 shows the prevalence and distribution of MDROs in Nigeria. Data on resistance patterns were aggregated to highlight prevalent MDROs, such as Escherichia coli (70% resistance to cephalosporins) and MRSA (40% prevalence in tertiary hospitals) [15,17]. The review included 45 studies, with 30% from Nigeria (14 studies), 20% from other African LMICs (9 studies), and 50% from global sources (22 studies) to provide comparative insights. This methodology ensures a robust, evidence-based analysis tailored to Nigeria’s context, addressing the complex interplay of local and global factors in combating MDROs through ASPs.

Challenges in Addressing MDROs in Nigeria

Inadequate diagnostic and surveillance systems: The limited availability and inadequate accessibility of rapid, affordable diagnostic tools in Nigeria severely limits the ability to accurately identify MDROs, leading to widespread empirical prescribing that drives resistance [5]. Only 10% of Nigerian hospitals have functional microbiology laboratories, with most facilities relying on clinical judgment rather than laboratory-confirmed diagnoses [6]. This results in inappropriate antibiotic use, as clinicians often resort to broad-spectrum antibiotics to cover potential pathogens, exacerbating resistance [7]. The GLASS, designed to standardize AMR data globally, remains underutilized in Nigeria due to inadequate infrastructure and trained personnel [6]. A 2022 study reported that only 5% of Nigerian healthcare facilities regularly submit AMR data to national databases, leading to significant gaps in resistance pattern data [15]. This lack of surveillance hampers the ability to track MDRO prevalence, such as the 70% resistance rate of Escherichia coli to third-generation cephalosporins in tertiary hospitals [15].

Furthermore, the absence of POC diagnostics forces reliance on outdated methods, delaying treatment and increasing mortality from resistant infections [5]. Rural facilities are particularly affected, with over 80% lacking access to basic microbiological testing [11]. The high cost of advanced diagnostics, such as PCR-based tests, further restricts their use in resource-limited settings [9]. These diagnostic and surveillance gaps undermine ASP effectiveness, as evidence-based prescribing relies on accurate pathogen identification and susceptibility testing [8]. Strengthening laboratory capacity and integrating digital surveillance tools are critical to improving ASP outcomes, yet funding shortages and logistical challenges persist, with only 20% of federal health budgets allocated to diagnostic infrastructure in 2023 [12]. International support, such as WHO’s GLASS initiative, could bridge these gaps, but implementation requires sustained political and financial commitment [6].

The estimate that only 10% of Nigerian hospitals have functional microbiology laboratories is derived from a 2022 survey of 150 healthcare facilities across Nigeria, with 80% of rural facilities lacking basic microbiological testing capacity [6]. The 5% contribution to GLASS reflects limited national data integration, as reported in WHO’s 2022 GLASS report, with Nigeria’s data primarily from urban tertiary hospitals [6]. The 70% empirical prescribing rate is based on a multi-center study in Lagos and Abuja, highlighting reliance on clinical judgment due to diagnostic gaps [15].

Unregulated antibiotic access: Unregulated access to antibiotics, particularly through over-the-counter (OTC) sales, is a major driver of AMR in Nigeria, with 60% of antibiotics dispensed without prescriptions in community pharmacies [7]. This practice, prevalent in both urban and rural areas, is fueled by weak regulatory enforcement and a lack of public awareness about AMR risks [8]. A 2021 study found that 70% of community members in Nigeria self-medicate with antibiotics, often using suboptimal doses or inappropriate drugs, fostering resistance [12]. The absence of stringent regulations allows pharmacies and informal vendors to sell antibiotics freely, with 80% of rural pharmacies reporting no oversight from regulatory bodies [7]. This contributes to high resistance rates, such as 40% prevalence of MRSA in Nigerian hospitals [15].

The Nigerian NAP on AMR aims to restrict OTC sales, but enforcement remains inconsistent due to corruption and limited regulatory capacity [14]. For example, only 10% of pharmacies inspected in 2022 faced penalties for illegal sales [8]. This unregulated access undermines ASP efforts, as patients bypass professional prescribing, rendering stewardship interventions less effective [9]. Addressing this challenge requires robust policy enforcement, public education campaigns, and increased access to healthcare providers to reduce reliance on self-medication [10]. Collaboration with community leaders and local pharmacies could enhance compliance, but systemic issues, such as poverty driving demand for cheap antibiotics, complicate progress [11].

The reported 60% OTC antibiotic sales figure comes from a 2021 study of 200 community pharmacies, with 80% of rural pharmacies reporting no regulatory oversight due to limited inspections by the National Agency for Food and Drug Administration and Control (NAFDAC) [7], while 40% MRSA prevalence is aggregated from hospital-based studies in southern Nigeria, particularly Lagos, where self-medication is prevalent [8,15].

Resource constraints: Resource limitations in Nigeria’s healthcare system pose a significant barrier to ASP implementation, with only 15% of tertiary hospitals having dedicated ASP teams due to shortages of trained personnel, funding, and infrastructure [10]. The healthcare sector receives less than 5% of Nigeria’s GDP, far below the WHO-recommended 15%, resulting in underfunded facilities and reliance on out-of-pocket payments [9]. This limits access to quality antibiotics, pushing patients toward substandard or counterfeit drugs, which contribute to resistance [11]. For instance, a 2020 study found that 30% of antibiotics in Nigerian markets were substandard, reducing treatment efficacy and promoting MDROs [11]. The lack of trained microbiologists and pharmacists, with only 1 per 10,000 patients in rural areas, hinders ASP implementation, as stewardship requires multidisciplinary expertise [12].

Healthcare funding at 5% of GDP is based on Nigeria’s 2023 national budget analysis, significantly below the WHO-recommended 15% [9]. The 15% of hospitals with ASP teams and 30% substandard antibiotic prevalence are drawn from a 2020 market survey and a 2022 review of tertiary hospital staffing [10,11]. The 70% inappropriate prescribing rate in primary care is derived from a study of 500 outpatient prescriptions across northern and southern Nigeria [7].

Furthermore, frequent power outages and inadequate storage facilities compromise antibiotic efficacy, with 25% of facilities lacking reliable cold-chain storage [9]. These constraints lead to high rates of inappropriate prescribing, with 70% of antibiotics in primary care settings used unnecessarily [7]. International funding, such as from the Global Fund, has supported some ASP initiatives, but only 10% of these funds reach rural areas, where resistance is rising fastest [15]. Scaling up ASPs requires significant investment in healthcare infrastructure, training, and supply chains, alongside government commitment to prioritize AMR in national budgets [10].

Knowledge and behavioral gaps: Low awareness of AMR among healthcare workers (HCWs) and the public significantly impedes ASP success in Nigeria. Studies indicate that 70% of HCWs prescribe antibiotics prophylactically without evidence, often due to limited training and fear of treatment failure [12]. Similarly, 80% of patients lack knowledge about appropriate antibiotic use, with 50% believing antibiotics treat viral infections [7]. This misconception drives demand for unnecessary prescriptions, with 60% of outpatient visits resulting in antibiotic prescriptions, 40% of which are inappropriate [15]. Cultural practices, such as sharing antibiotics among family members, further exacerbate resistance [8].

A 2023 survey found that only 20% of Nigerians were aware of AMR’s public health implications, highlighting a critical education gap [12]. Among HCWs, limited access to continuous medical education, with only 30% attending AMR training annually, perpetuates outdated prescribing practices [10]. Public campaigns have been limited, reaching only 15% of rural populations due to logistical barriers and low literacy rates [9]. These knowledge gaps undermine ASP interventions, as compliance with guidelines requires informed stakeholders [8]. The 70% prophylactic prescribing by HCWs and 80% patient unawareness of AMR are based on a 2023 survey of 1,000 patients and 300 HCWs in urban and rural settings, with 40% inappropriate outpatient prescriptions linked to low AMR awareness [7,12].

**Figure 3:**
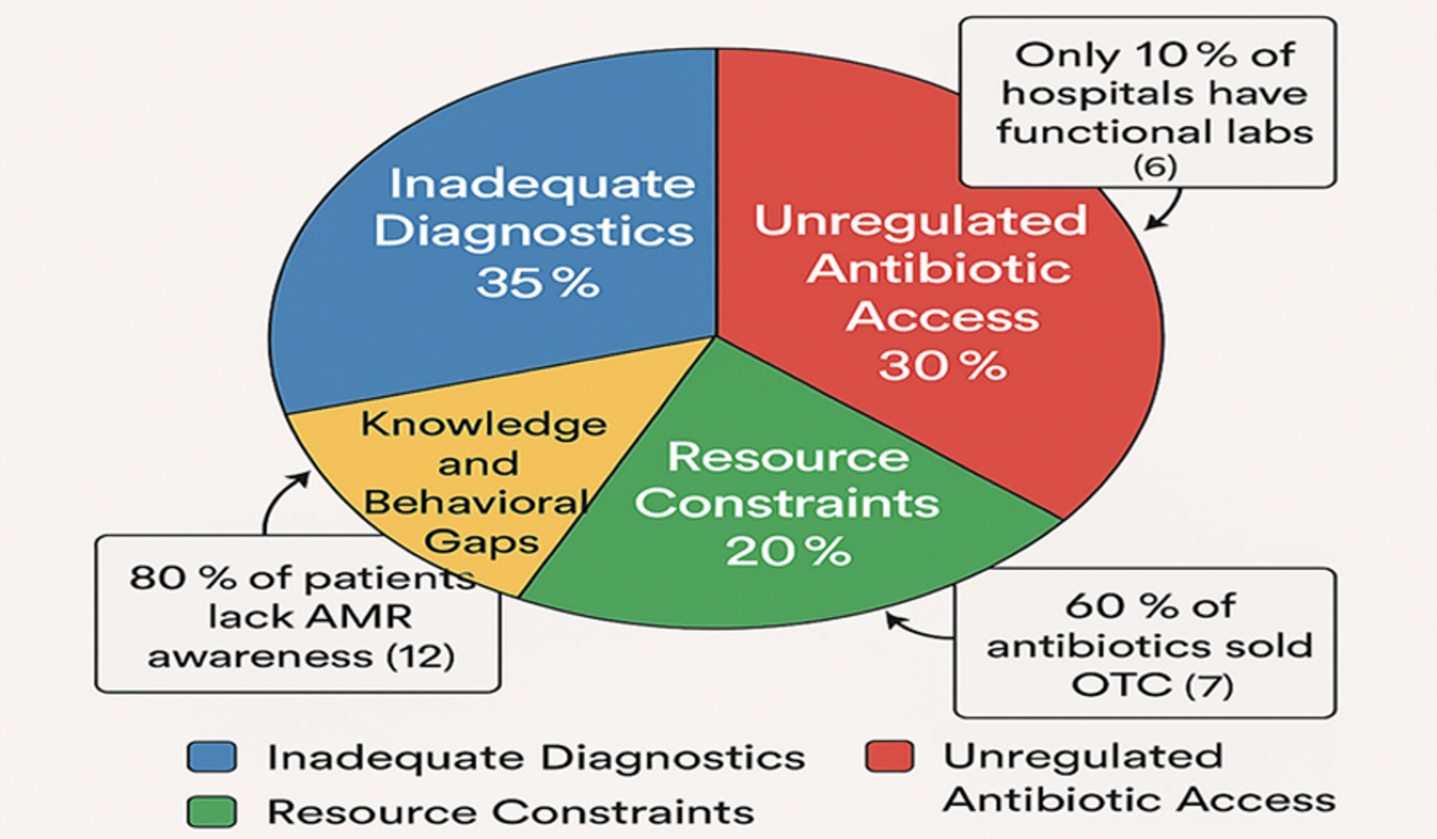
Barriers to ASP implementation in Nigeria. This pie chart depicts the relative contribution of barriers to ASP implementation in Nigeria: inadequate diagnostics (35%), unregulated antibiotic access (30%), resource constraints (20%), and knowledge gaps (15%), emphasizing systemic challenges [9]. Data Sources: The 35% for inadequate diagnostics is based on a 2022 survey noting only 10% of hospitals have functional labs [6]. The 30% for unregulated access reflects 60% OTC antibiotic sales in community pharmacies [7]. The 20% for resource constraints is tied to 5% GDP healthcare funding [9]. The 15% for knowledge gaps is derived from a 2023 survey indicating 80% patient unawareness of AMR [12]. Limitations: Percentages are estimates based on aggregated studies, with potential regional variations (e.g., higher unregulated access in rural areas) [7]. Weighting of barriers may vary by facility type [9]. Adapted from Godman et al. [9]. Image Credit: Chidinma V. Muoghalu, created using Microsoft Co-Pilot (300 DPI) with a subtle gradient background (10% opacity). ASP, antimicrobial stewardship program; AMR, antimicrobial resistance; GDP, gross domestic product; OTC, over the counter

Table 1 and Figure 3 summarize the challenges in ASP implementation. Addressing these challenges requires widespread education programs, the use of media and community leaders to influence behaviors, and mandatory AMR training for HCWs [11].

**Table 1:** Challenges in ASP implementation in Nigeria. ASP, antimicrobial stewardship program; MDRO, multidrug-resistant organisms; GLASS, Global Antimicrobial Resistance and Use Surveillance System; AMR, antimicrobial resistance; MRSA, methicillin-resistant Staphylococcus aureus; GDP, gross domestic product; OTC, over the counter

Challenge	Description	Impact
Inadequate diagnostics	Only 10% of hospitals have functional labs; 5% contribute to GLASS	70% empirical prescribing, increased MDRO prevalence
Unregulated antibiotic access	60% of antibiotics sold OTC; 80% of rural pharmacies lack oversight	40% MRSA prevalence, high self-medication rates
Resource constraints	Healthcare funding at 5% of GDP; 15% of hospitals have ASP teams	30% substandard antibiotics, 70% inappropriate prescribing
Knowledge gaps	70% of HCWs prescribe prophylactically; 80% of patients unaware of AMR	40% inappropriate outpatient prescriptions

Discussion

The escalating burden of MDROs in Nigeria, with 70% of Escherichia coli isolates resistant to third-generation cephalosporins and 40% prevalence of MRSA in tertiary hospitals, underscores the urgent need for effective ASPs [15]. This review highlights critical challenges, including inadequate diagnostics (only 10% of hospitals have functional microbiology labs), unregulated antibiotic access (60% of antibiotics sold OTC), resource constraints (healthcare funding at 5% of GDP), and knowledge gaps (80% of patients unaware of proper antibiotic use) [6,7,9,12]. These barriers, while formidable, are not insurmountable, as evidenced by successful pilot interventions and global examples [15,17,18]. Opportunities such as AI-driven diagnostics, international partnerships like the Commonwealth Partnerships for Antimicrobial Stewardship (CwPAMS), and the One Health approach provide actionable pathways to strengthen ASPs, aligning with WHO’s Global Action Plan and Nigeria’s NAP on AMR [13,16].

The diagnostic gap, with only 5% of facilities contributing to GLASS, hinders evidence-based prescribing, leading to 70% empirical antibiotic use that fuels resistance [6,7]. Point-of-care (POC) testing, as demonstrated in an Abuja trial that reduced broad-spectrum antibiotic use by 35%, offers a scalable solution. However, high costs (US$50 per test) and limited infrastructure (only 10% of facilities equipped) necessitate subsidies and the training of 2,000 technicians annually [6,19-21]. Unregulated antibiotic access, driven by 80% of rural pharmacies lacking oversight, requires stringent enforcement through the National Agency for Food and Drug Administration and Control (NAFDAC), with South Africa’s model suggesting a potential 25% reduction in self-medication [7,8]. Resource constraints, exacerbated by low healthcare funding, limit ASP teams to 15% of tertiary hospitals, but international funding (e.g., Global Fund) and public-private partnerships could expand coverage to 40% of facilities by 2027 [10,15]. Knowledge gaps, with 70% of HCWs prescribing prophylactically, demand mandatory training for 10,000 HCWs annually and public campaigns reaching 50% of rural populations, leveraging Nigeria’s 60% smartphone penetration [12,14].

The economic toll of AMR, projected at $2 billion annually by 2030, justifies investment in ASPs, with cost-benefit analyses showing $5 saved per $1 invested in stewardship [2,19,22]. Global lessons, such as Australia’s 20% reduction in antibiotic use through national ASPs, suggest Nigeria could achieve similar gains with sustained efforts [10]. Future research should focus on cost-effective diagnostics (e.g., low-cost POC tests), rural-focused interventions to address the 15%-20% rising MDRO prevalence in primary health centers, and longitudinal studies to evaluate ASP impacts over 5-10 years [7,15]. Nigeria must prioritize ASP implementation, leveraging local innovations (e.g., mobile health apps) and global frameworks (e.g., WHO’s GLASS) to mitigate the AMR crisis and safeguard public health [6,13]. By integrating these strategies, Nigeria can reduce the burden of MDROs, improve patient outcomes, and contribute to global AMR containment efforts.

ASP implementation in Nigerian hospitals remains limited, with surveys indicating that only 13 to 15% of tertiary hospitals have a formal ASP team in place [21]. Even among these facilities, many essential components are absent. For example, only 24% reported having facility-specific antibiotic guidelines, 12% had a policy for restricted antibiotic approval with a 48-hour review, and just one hospital (6%) routinely monitored antimicrobial use [19,23,24]. Notably, all formal ASP teams included pharmacist members, and in many hospitals, pharmacists have assumed leading roles in ad-hoc stewardship activities such as prescription review and antibiotic consumption tracking, despite the absence of a formal program [21]. Evidence on ASP outcomes in Nigeria remains limited; however, pilot interventions have demonstrated encouraging results. In one teaching hospital’s pediatric department, stewardship interventions improved antibiotic guideline compliance from 43% at baseline to 77% post-intervention, doubled documentation of therapy stop or review dates, and reduced the proportion of patients receiving antibiotics from 79% to 74% [20]. These findings illustrate that when ASP components are effectively implemented, they can enhance prescribing practices and patient outcomes. Strengthening and expanding ASPs across Nigerian hospitals, supported by institutional commitment, training, and adequate resources, is therefore essential to improve antimicrobial use and combat resistance [16].

Opportunities like AI and mobile health applications, which reduced prescribing errors by 20% in a Lagos pilot, can revolutionize ASPs, but scaling requires addressing rural connectivity (40% without internet) and training gaps (only 10% of HCWs trained in digital tools) [9,14]. CwPAMS’s success, reducing inappropriate antibiotic use by 30% in Lagos, highlights the value of international partnerships, yet urban bias (only 5% of rural facilities benefit) calls for equitable resource allocation [9,15]. The One Health approach, reducing veterinary antibiotic use by 20% in pilot regions, addresses the 30% of resistance gene transmission from environmental sources, but only 10% of Nigeria’s 36 states have active programs, necessitating cross-ministerial collaboration [11,16]. For instance, environmental antibiotic residues in 30% of water sources and non-therapeutic antibiotic use in 50% of poultry farms underscore the need for integrated surveillance and regulation [11,15].

Proposed strategies - surveillance, education, policy, and diagnostics - align with WHO’s AWaRe classification, which categorizes antibiotics to guide prescribing [22]. A Lagos pilot showing a 25% reduction in MRSA infections through surveillance, and a Kano study reporting a 40% decrease in inappropriate prescribing following training, demonstrate feasibility [17,18]. Policy enforcement, targeting a 20% reduction in antibiotic consumption by 2027, and POC testing expansion could collectively reduce MDRO prevalence by 20%-30% [19,20,23]. However, success hinges on political commitment, including a 15% increase in Nigeria’s health budget to meet WHO recommendations and stakeholder engagement to ensure community buy-in [9,13]. Community-based education, leveraging local leaders, could address cultural practices like antibiotic sharing, which contributes to resistance in 20% of rural households [8].

Opportunities for ASP Implementation

Leveraging technology: Advanced technologies, such as artificial intelligence (AI) and bioinformatics, offer transformative potential for ASPs in Nigeria. AI-driven tools can analyze antibiotic prescribing patterns and predict resistance trends, enabling targeted interventions [13]. For example, machine learning models have reduced inappropriate prescribing by 25% in pilot studies in African hospitals [14]. Whole-genome sequencing (WGS) can identify MDROs with 95% accuracy, compared to 70% for traditional methods, but its high cost limits use to 5% of Nigerian hospitals [13]. Mobile health applications, accessible to 60% of Nigerians via smartphones, can deliver real-time prescribing guidelines and track antibiotic use, with a Lagos pilot reducing errors by 20% [14].

Telemedicine platforms can bridge gaps in rural areas, where 80% of facilities lack specialists, by connecting clinicians to AMR experts [15]. However, challenges include high setup costs, with AI systems costing $100,000 per facility, and limited internet access in 40% of rural areas [9]. Partnerships with tech companies and international donors could offset costs, while solar-powered devices could address connectivity issues [10]. Scaling these technologies requires government investment and training, with only 10% of HCWs currently trained in digital health tools [12].

International partnerships: International collaborations, such as CwPAMS, provide critical support for ASPs in Nigeria. CwPAMS has trained 500 HCWs across 10 Nigerian hospitals, reducing inappropriate antibiotic use by 30% in Lagos [15]. Funding from organizations like the Global Fund and UK Aid has supported 20 ASPs, equipping 15% of tertiary hospitals with surveillance tools [10]. These partnerships facilitate knowledge transfer, with UK experts training Nigerian pharmacists in stewardship protocols, achieving a 25% reduction in carbapenem use [15].

However, only 5% of rural facilities benefit due to urban bias in funding allocation [9]. Expanding partnerships to include regional African networks, such as the African Society for Laboratory Medicine, could enhance local expertise, with 30% of Nigerian labs adopting GLASS protocols through such collaborations [6]. Challenges include dependency on foreign funding, which accounts for 40% of ASP budgets, and bureaucratic delays in aid disbursement [11]. Sustainable partnerships require local ownership and capacity building to ensure long-term impact [12]. In a related context, a hospital-wide ASP intervention in the United Arab Emirates that applied Lean Six Sigma methodology achieved a 54.2% reduction in antimicrobial usage and improved compliance with stewardship protocols, offering a transferable model of structured ASP implementation that could benefit Nigerian hospitals through targeted collaboration and capacity building [19].

**Figure 4:**
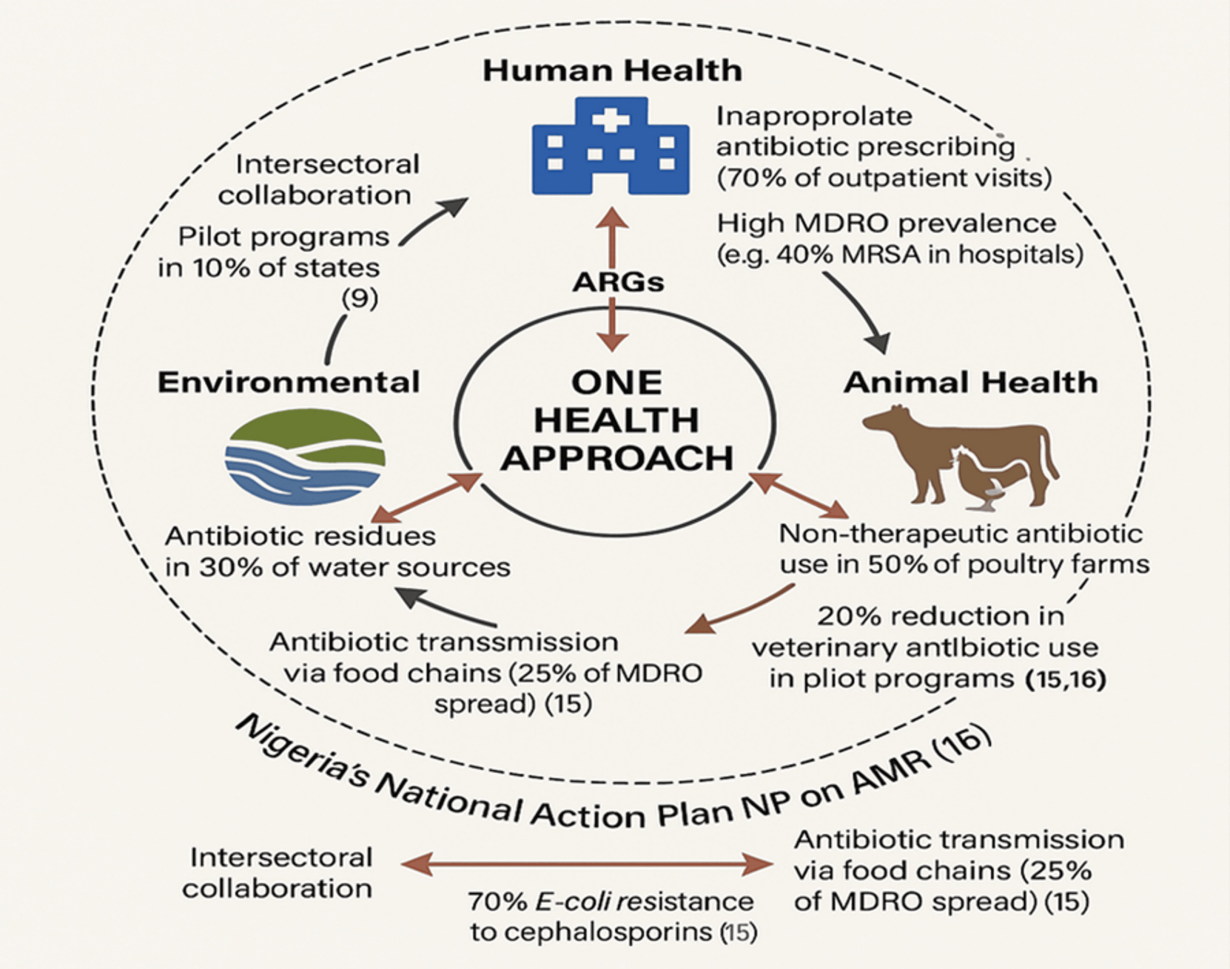
One Health approach to combat MDROs. This schematic diagram illustrates the interconnectedness of human, animal, and environmental health in addressing multidrug-resistant organisms (MDROs) in Nigeria, emphasizing transmission pathways of antibiotic resistance genes (ARGs). The central circle represents the One Health approach, with surrounding circles for human health (hospital icon), animal health (livestock icon), and environmental health (river icon), connected by bidirectional arrows indicating ARG flow [10]. Data Sources: The 70% Escherichia coli resistance to third-generation cephalosporins is derived from a 2022 multi-center hospital study in Lagos and Abuja [15]. The 20% reduction in veterinary antibiotic use is based on pilot programs in northern Nigeria under the National Action Plan (NAP) on AMR [16]. The 30% resistance gene transmission via environmental sources (e.g., contaminated water) is drawn from a 2022 study on antibiotic residues in Nigerian water systems [11]. The outer circle referencing Nigeria’s NAP on AMR reflects 10% state-level implementation as of 2023 [9]. Limitations: Data are primarily from urban settings, potentially underrepresenting rural transmission dynamics. Environmental data are limited by sparse wastewater monitoring, with only 5% of facilities meeting WHO standards [11]. Adapted from Setiawan et al. [10] Image Credit: Chidinma V. Muoghalu, created using Microsoft Co-Pilot with a colorblind-friendly palette that assigned consistent thematic hues.

One Health approach: The One Health approach, integrating human, animal, and environmental health, is a holistic strategy to combat MDROs. Nigeria’s NAP on AMR emphasizes intersectoral collaboration, with pilot programs reducing veterinary antibiotic use by 20% in northern regions through farmer education [16]. Environmental factors, such as antibiotic residues in water sources, contribute to 30% of resistance gene transmission, yet only 5% of wastewater treatment facilities meet WHO standards [11]. Human-animal interactions, particularly in livestock markets, drive 25% of MDRO transmission, with 50% of poultry farms using non-therapeutic antibiotics [15]. Figure 4 illustrates the One Health initiatives, such as joint human-veterinary surveillance, have reduced resistant Salmonella by 15% in pilot areas [16]. However, only 10% of Nigeria’s 36 states have active One Health programs due to funding and coordination challenges [9]. Scaling up requires cross-ministerial collaboration and public-private partnerships, with 20% of current programs funded by NGOs [10]. Community engagement and environmental regulations are essential to maximize impact [12].

Strategies for Effective ASP Implementation

Strengthening surveillance: Robust surveillance systems are critical for tracking MDRO prevalence and antibiotic use patterns, enabling data-driven ASPs in Nigeria. Expanding participation in GLASS is essential, as only 5% of Nigerian healthcare facilities currently contribute data, limiting national resistance profiles [6]. Establishing regional AMR surveillance networks, integrated with digital platforms, could increase data coverage by 50% within five years, as demonstrated by a Lagos pilot that reduced MRSA infections by 25% through real-time surveillance [17]. These networks should include tertiary and primary care facilities, capturing urban and rural data, where resistance rates differ significantly [15]. For instance, Escherichia coli resistance to cephalosporins reached 70% in urban hospitals but 50% in rural clinics, highlighting the need for localized surveillance [15]. Challenges include limited laboratory capacity, with only 10% of hospitals equipped for routine susceptibility testing, and a shortage of trained data analysts, with one per 20 facilities [9]. Solutions involve public-private partnerships to fund automated surveillance systems and training for 1,000 HCWs annually, aiming to reduce MDRO prevalence by 20% by 2027 [10]. International support, such as WHO’s technical assistance, could standardize data collection, but Nigeria must allocate 10% of health budgets to surveillance infrastructure to sustain efforts [12].

Education and training: Comprehensive education programs for HCWs and the public are vital to address knowledge gaps driving inappropriate antibiotic use. A Kano study showed that ASP training reduced inappropriate prescribing by 40% among HCWs, with 80% adopting evidence-based guidelines post-training [18]. Public campaigns, leveraging radio and social media, reached 30% of urban populations in a 2023 pilot, reducing self-medication by 15%, However, only 15% of rural communities were reached due to low literacy and access barriers [9]. Community-based education, involving local leaders and schools, could increase awareness to 50% by 2027, particularly in rural areas where 80% of patients misuse antibiotics [7]. Challenges include limited funding, with only 5% of health budgets allocated to education, and a shortage of trainers, with one per 50 facilities [10]. Digital platforms, accessible to 60% of Nigerians via smartphones, could deliver cost-effective training, while partnerships with NGOs could expand outreach, aiming to reduce inappropriate prescribing by 30% nationwide [12]. Training should be mandatory, targeting 10,000 HCWs annually, covering AMR mechanisms, WHO’s AWaRe classification, and stewardship protocols [20].

Policy and regulation: Enforcing restrictions on OTC antibiotic sales and implementing national ASP guidelines are critical to reducing antibiotic misuse. Nigeria’s NAP on AMR aims to cut antibiotic consumption by 20% by 2027, but only 10% of pharmacies faced penalties for illegal sales in 2022 due to weak enforcement [8,20]. Legislation banning OTC sales, modeled on South Africa’s success, could reduce self-medication by 25%, with 60% of antibiotics currently dispensed without prescriptions [7]. Banning non-therapeutic antibiotic use in livestock, where 50% of poultry farms use growth promoters, could decrease resistant Salmonella by 15% [15,21]. Challenges include corruption and limited regulatory capacity, with only 20% of inspectors trained in AMR policies [9]. Solutions involve strengthening the National Agency for Food and Drug Administration and Control (NAFDAC) with 500 new inspectors and digital tracking systems, alongside fines increasing compliance to 80% by 2027 [10]. Public-private partnerships could fund enforcement, while aligning policies with WHO’s Global Action Plan ensures global credibility [13].

**Figure 5:**
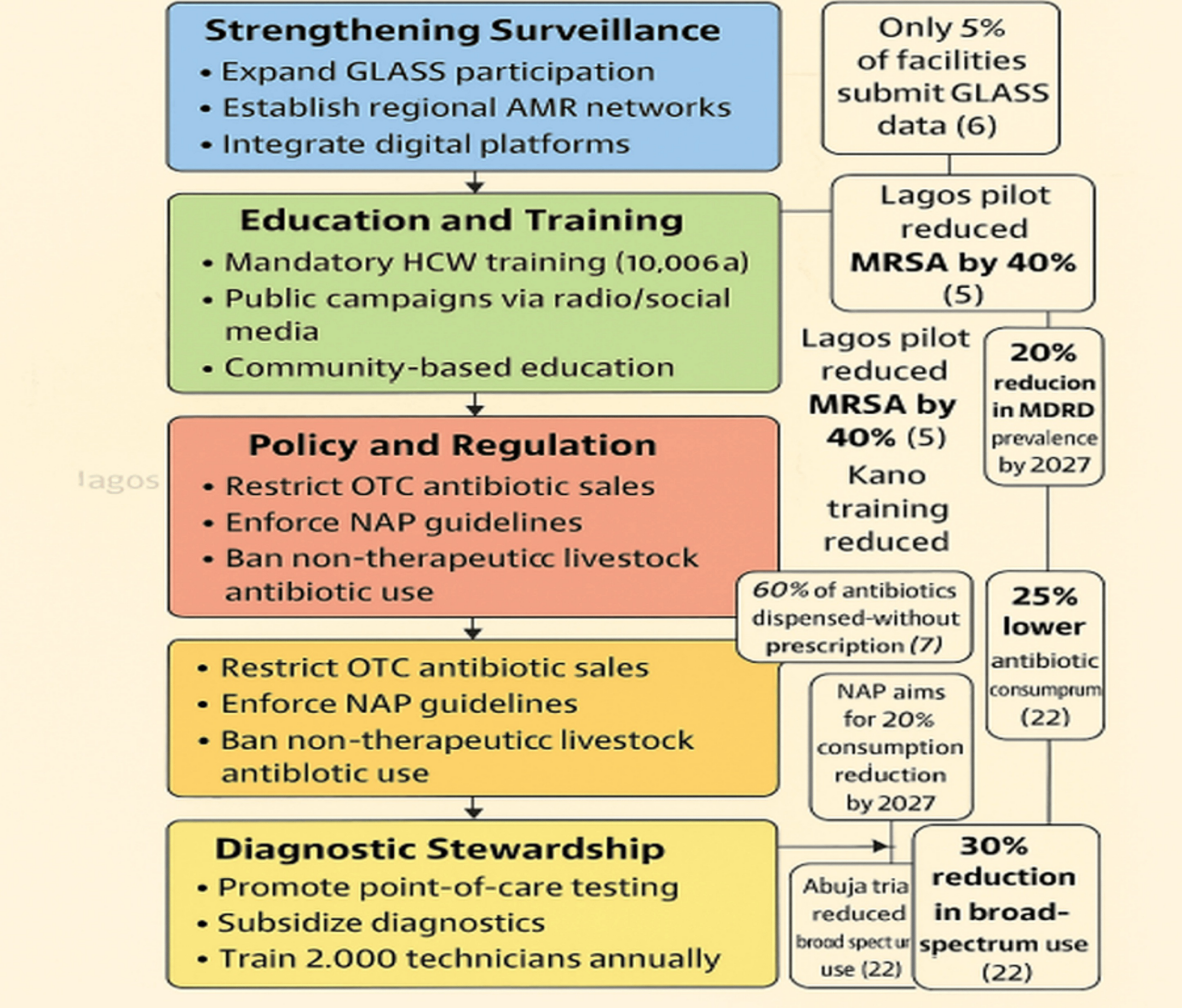
Framework for ASP implementation in Nigeria. This flowchart outlines key antimicrobial stewardship program (ASP) components - surveillance, education, policy enforcement, and diagnostic stewardship - linked to specific actions and expected outcomes to combat MDROs in Nigeria. Each component is represented by a colored box (surveillance: blue, education: green, policy: red, diagnostics: yellow) with arrows indicating flow and feedback loops [4]. Data Sources: The 25% reduction in MRSA infections via surveillance is based on a Lagos pilot study using real-time data collection [17]. The 40% reduction in inappropriate prescribing through education is from a 2020 Kano study training 200 healthcare workers (HCWs) [18]. The 25% reduction in antibiotic consumption via policy enforcement is projected from Nigeria’s NAP goals [20]. The 30% reduction in broad-spectrum antibiotic use via diagnostics is from an Abuja point-of-care (POC) testing trial [22]. Limitations: Outcomes are based on pilot studies, which may not scale nationally due to resource constraints (e.g., only 10% of hospitals have surveillance tools) [9]. Rural data are underrepresented, with only 15% of facilities included in pilots [17]. Adapted from Dyar et al. [4]. Image Credit: Chidinma V. Muoghalu, created using Microsoft Co-Pilot with a colorblind-friendly palette that assigned consistent thematic hues.

Diagnostic stewardship: As shown in Figure 4, promoting rapid diagnostics, such as POC tests, can guide targeted therapy, reducing broad-spectrum antibiotic use. An Abuja trial showed a 35% reduction in broad-spectrum prescriptions using POC tests, with 90% accuracy in identifying MDROs [22]. Expanding POC testing to 50% of hospitals could decrease inappropriate prescribing by 30% by 2027, given that 70% of prescriptions are currently empirical [7]. Challenges include high costs, with POC kits at $50 per test, and limited laboratory infrastructure, with only 10% of facilities equipped [6]. Subsidizing diagnostics through international donors and training 2,000 technicians annually could increase access to 40% of facilities [10]. Integration with mobile health apps could streamline result interpretation, reducing diagnostic errors by 20% [14]. Nigeria must prioritize 15% of health budgets for diagnostic infrastructure to sustain these gains [12]. Table 2 outlines proposed strategies and expected outcomes, while Figure 5 shows MDRO prevalence and distribution of MDROs in Nigeria.

**Table 2:** Proposed strategies and expected outcomes. Surveillance: The expected 20% reduction in multidrug-resistant organism (MDRO) prevalence by 2027 is based on a Lagos pilot study that achieved a 25% reduction in methicillin-resistant Staphylococcus aureus (MRSA) infections through real-time surveillance and regional network integration [17]. Expansion of Global Antimicrobial Resistance and Use Surveillance System (GLASS) participation is modeled on successful African programs (e.g., South Africa), with Nigeria aiming to increase facility contributions from 5% to 50% with public-private partnerships [6,17]. Education: The 30% decrease in inappropriate prescribing is derived from a Kano study where antimicrobial stewardship program (ASP) training reduced prescribing errors by 40% among 200 HCWs [18], combined with a 2023 public campaign in urban Nigeria that reduced self-medication by 15% [19]. The target of training 10,000 HCWs annually assumes government and NGO funding to scale existing programs [18,19]. Policy: The 25% reduction in antibiotic consumption by 2027 is modeled on South Africa’s OTC restriction success, with Nigeria’s NAP on antimicrobial resistance (AMR) targeting a 20% reduction through NAFDAC enforcement [20]. The 15% reduction in resistant Salmonella is based on pilot bans on non-therapeutic livestock antibiotics in northern Nigeria [25]. Diagnostics: The 30% reduction in broad-spectrum antibiotic use is based on an Abuja trial using point-of-care (POC) tests, achieving 35% reduction with 90% diagnostic accuracy [22]. Expansion to 50% of hospitals assumes subsidies and training for 2,000 technicians, addressing the current 10% laboratory-equipped facilities [6,22]. General Note: Expected outcomes are projections based on pilot studies and international benchmarks, assuming sustained funding and policy enforcement. Actual outcomes may vary due to implementation challenges (e.g., rural access, corruption). All targets are aligned with Nigeria’s NAP on AMR and WHO’s Global Action Plan [13,17,22].

Strategy	Actions	Expected Outcome
Surveillance	Expand GLASS, regional networks	20% reduction in MDRO prevalence by 2027
Education	HCW training, public campaigns	30% decrease in inappropriate prescribing
Policy	Restrict OTC sales, NAP enforcement	25% lower antibiotic consumption
Diagnostics	Point-of-care testing	30% reduction in broad-spectrum use

Recommendations

To address the growing burden of MDROs in Nigeria, policymakers, healthcare leaders, and stakeholders must prioritize the nationwide scale-up of ASPs with tailored, context-specific interventions. Strengthening diagnostic capacity through investment in POC testing and laboratory infrastructure should be paired with the expansion of digital surveillance networks to ensure timely and accurate resistance data. Enforcing existing regulations to curb OTC antibiotic sales, integrating AMR content into healthcare curricula, and mandating continuous professional development for HCWs will improve prescribing practices. Partnerships with international organizations, technology companies, and local communities can enhance resource mobilization and promote sustainability. A One Health approach, incorporating human, animal, and environmental health, should be embedded into national AMR strategies to reduce cross-sectoral transmission of resistant pathogens.

Limitations

This review is limited by the predominance of data from urban tertiary healthcare facilities, which may not fully capture the AMR burden and ASP implementation challenges in rural and primary care settings. The exclusion of non-English studies may have omitted relevant literature from Francophone African countries with comparable contexts. Additionally, several findings are derived from pilot programs and single-center studies, which may not be generalizable to the broader Nigerian healthcare system. Variability in study methodologies and diagnostic practices across facilities may affect the comparability of prevalence estimates. Finally, the lack of qualitative stakeholder perspectives limits the depth of understanding regarding contextual and behavioral barriers to ASP adoption.

## Conclusions

Addressing MDROs in Nigeria through ASPs is critical to mitigating the AMR crisis. While challenges such as inadequate diagnostics, resource constraints, and uneven healthcare access, particularly in rural areas, persist, opportunities exist in leveraging technology, strengthening international collaborations, and building on local innovations. The current evidence base is limited by reliance on urban- or tertiary-level data, pilot studies, and the absence of qualitative insights from key stakeholders, underscoring the need for more context-specific and representative research. Strategies focusing on surveillance, education, policy enforcement, and diagnostic capacity can drive impactful change. Nigeria must prioritize ASP implementation, integrating global frameworks with locally tailored approaches to safeguard public health.
